# A 16-Week Aerobic Exercise Intervention Does Not Affect Hippocampal Volume and Cortical Thickness in Mild to Moderate Alzheimer’s Disease

**DOI:** 10.3389/fnagi.2018.00293

**Published:** 2018-09-25

**Authors:** Kristian Steen Frederiksen, Christian Thode Larsen, Steen Gregers Hasselbalch, Anders Nymark Christensen, Peter Høgh, Lene Wermuth, Birgitte Bo Andersen, Hartwig Roman Siebner, Ellen Garde

**Affiliations:** ^1^Danish Dementia Research Centre, Department of Neurology, Rigshospitalet, University of Copenhagen, Copenhagen, Denmark; ^2^Danish Research Centre for Magnetic Resonance, Copenhagen University Hospital Hvidovre, Hvidovre, Denmark; ^3^Department of Applied Mathematics and Computer Science, Technical University of Denmark, Kongens Lyngby, Denmark; ^4^Regional Dementia Research Center, Department of Neurology, Zealand University Hospital, Roskilde, Denmark; ^5^Department of Clinical Medicine, University of Copenhagen, Copenhagen, Denmark; ^6^Dementia Clinic, Odense University Hospital, Odense, Denmark; ^7^Department of Neurology, Bispebjerg Hospital, Copenhagen University Hospital, Copenhagen, Denmark

**Keywords:** Alzheimer’s disease, physical exercise, aerobic exercise, MRI, dementia, hippocampus, cortical thickness, intervention

## Abstract

**Introduction:** Brain imaging studies in healthy elderly subjects suggest a positive effect of aerobic exercise on both brain structure and function, while the effects of aerobic exercise in Alzheimer’s Disease (AD) has been scarcely investigated.

**Methods:** In a single-blinded randomized MRI study, we assessed the effects of an aerobic exercise intervention on brain volume as measured by magnetic resonance imaging (MRI) and its correlation to cognitive functioning in patients with AD. The study was a sub-study of a larger randomized controlled trial (ADEX study). Forty-one patients were assigned to a control or exercise group. The exercise group performed 60-min of aerobic exercise three times per week for 16 weeks. All participants underwent whole-brain MRI at 3 Tesla and cognitive assessment at baseline and after 16 weeks. Attendance and intensity were monitored providing a total exercise load. Changes in regional brain volumes and cortical thickness were analyzed using Freesurfer software.

**Results:** There was no effect of the type of intervention on MRI-derived brain volumes. In the entire group with and without training, Exercise load showed a positive correlation with changes in volume in the hippocampus, as well as frontal cortical thickness. Volume changes in frontal cortical thickness correlated with changes in measures of mental speed and attention and exercise load in the exercise group.

**Conclusion:** We did not find evidence to support an effect of 16 weeks of aerobic exercise on brain volume changes in patients with AD. Longer intervention periods may be needed to affect brain structure as measured with volumetric MRI.

**Clinical Trial registration:** ClinicalTrials.gov Identifier: NCT01681602, registered September 10th, 2012 (Retrospectively registered).

## Introduction

Observational studies have found a physically active lifestyle to be associated with a reduced risk for later cognitive decline including progression to dementia (Heyn et al., 2004; Aarsland et al., 2010), and accumulation of cerebral beta-amyloid and tau (Liang et al., 2010; Okonkwo et al., 2014; Merrill et al., 2016). Several randomized controlled trials (RCTs) of exercise interventions have demonstrated a positive effect on cognition in mild cognitive impairment (MCI) (Baker et al., 2010a; Suzuki et al., 2013) and Alzheimer’s disease (AD) (Venturelli et al., 2011; Vreugdenhil et al., 2012; Hoffmann et al., 2015). In a recent RCT in patients with mild-to-moderate AD, we found a positive effect of a 16-week aerobic exercise intervention on physical fitness, neuropsychiatric symptoms, and, in a subgroup, an effect on mental speed and attention (Hoffmann et al., 2015; Sobol et al., 2016). These findings highlight the fact that a relatively short exercise intervention is able to impact factors important for patient wellbeing.

Evidence for the biological mechanisms underlying a relationship between brain and exercise remains sparse. Animal studies, which have aimed to elucidate such mechanisms indicate several different pathways to be involved (Kramer et al., 2006; van Praag, 2008, 2009). This includes induction of neurogenesis in the hippocampus (van Praag et al., 1999; Intlekofer and Cotman, 2013), and anti-inflammatory changes (Ryan and Kelly, 2016). Similarly, pathology such as beta-amyloid and tau accumulation have been shown to be attenuated by exercise in animal models (Adlard et al., 2005; Yuede et al., 2009; Kang and Cho, 2015; Moore et al., 2016). In humans, exercise may have an effect on the hippocampus in healthy elderly subjects (Erickson et al., 2009, 2011) and MCI (ten Brinke et al., 2015), although results have been conflicting (Colcombe et al., 2006; Niemann et al., 2014; Tamura et al., 2015; Wagner et al., 2015). Other brain regions, most consistently frontal areas, have also been reported to be affected by exercise in healthy older subjects (Taubert et al., 2011; Scheewe et al., 2013; Reiter et al., 2015; Tamura et al., 2015; Köbe et al., 2016).

Hippocampal atrophy is a pathological hallmark of AD, but is also found in other neurodegenerative diseases (Toyoshima et al., 2003; Whitwell et al., 2011; Abdulla et al., 2014), healthy aging (Chowdhury et al., 2011), and depression (Frodl et al., 2002). Moreover, in patients with MCI where biomarkers confirming the presence of AD pathology are not available the underlying cause may include many different etiologies. Hence, hippocampal atrophy in healthy aging and MCI may be driven by a plethora of pathological processes. An effect of exercise on the hippocampus in healthy subjects and MCI may therefore not directly translate to patients in the dementia stage of AD pathology. As follows, studies of exercise interventions in AD are needed to test whether hippocampal atrophy in AD may be modifiable by exercise. In a single study, a mixed patient population consisting of both MCI and AD patients underwent an exercise intervention of 26 weeks, in which hippocampal atrophy rates did not differ between groups (Morris et al., 2017).

The paucity of data prompted the present study with the primary objective to test whether an aerobic exercise intervention attenuates hippocampal atrophy in patients with mild dementia due to AD. Furthermore, we investigated whether brain atrophy in other brain regions may be slowed down by exercise, in AD. Lastly, we examined correlations between changes in brain volume and exercise attendance, physical fitness and cognitive measures.

## Materials and Methods

### Participants and Study Design

**FIGURE 1:**
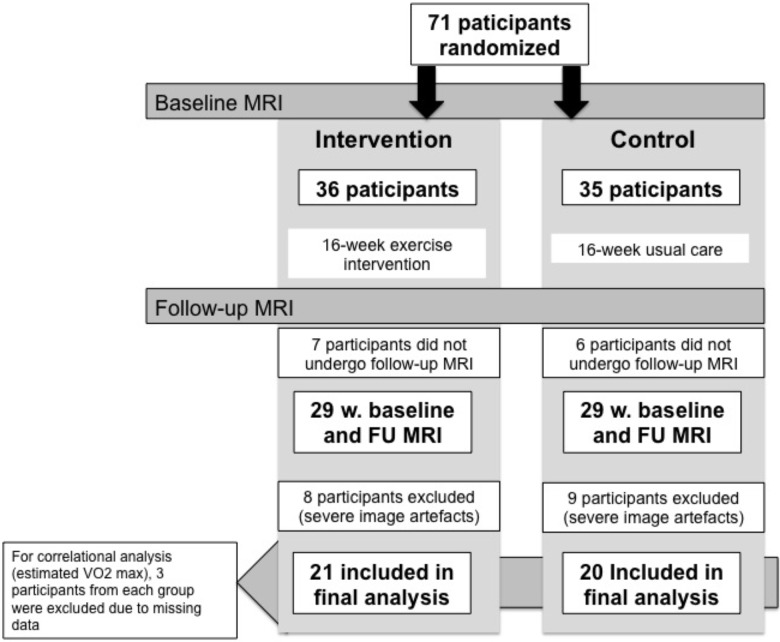
Flow diagram of participant flow through the study.

Preserving Cognition, Quality of Life, Physical Health and Functional Ability in AD: The Effect of Physical Exercise (ADEX) study is a multicenter single-blinded randomized study of moderate-to-high intensity exercise in patients with mild to moderate AD. The primary objective was to assess the effects of moderate-to-high intensity exercise on cognitive and physical functioning, neuropsychiatric symptoms, quality of life and Activities of daily living (ADL). A total of 200 participants were included. Participants were randomly assigned to either the intervention arm, consisting of three weekly sessions of 60 min of moderate-to-high-intensity aerobic exercise for 16 weeks, or control arm, consisting of usual care, in a blinded manner. Assessment of cognitive function, ADL, neuropsychiatric symptoms, blood sampling and physical function was carried out before and after intervention for all participants. Assessment was carried out by blinded assessors. Inclusion criteria included: (1) AD according to NINCDS-ADRDA and DSM-IV criteria, (2) age between 50 and 90 years, (3) Mini Mental State Examination (MMSE) score of more than 19, (4) at least 3 months of stable doses if the patient was receiving anti-dementia medication or mood stabilizing medication, (5) informed consent. Exclusion criteria included (1) severe psychiatric illness, (2) alcohol or drug abuse within the last 2 years, (3) participation in aerobic exercise (moderate-to-hard intensity) more than twice weekly on a regular basis, and (4) any medical condition which precluded engagement in the exercise program (e.g., severe neurological or medical illness, presence of several cardiovascular risk factors). Detailed description of rationale and design may be found elsewhere (Hoffmann et al., 2013) as well as the principle findings (Hoffmann et al., 2015; Sobol et al., 2016). The present study reports results from a MRI substudy within the ADEX study. Seventy-one participants from three centers underwent brain MRI at baseline. Thirteen participants dropped out of the study, and did not undergo MRI at 16-week follow-up, and 17 patients were excluded from further analysis of MRI due to poor MRI data quality which did not enable imaging processing, leaving 41 participants for the present study. For correlations between brain volume and estimated VO2 max, an additional six subjects were excluded due to missing data on estimated VO2 max. See **Figure 1** for flow diagram.

The ADEX trial was approved by the The Committees of Biomedical Research Ethics for the Capital Region (Protocol no.: H-3-2011-128) and by the Danish Data Protection Agency (j.no.: 30-0718). Both approvals were given, in accord with Danish national legislation, as umbrella approvals which covered any and all centers involved.

### MRI Acquisition

Both baseline and follow-up MRI was performed using a 3.0-T Siemens Trio scanner and included T1-weighed magnetization-prepared rapid gradient echo (MPRAGE) (TE 3.04 ms, TR 1550 ms, FoV read 256 mm, FoV phase 100%, 192 slices), T2-weighed fast spin echo (TE 354 ms, TR 3000 ms, FoV read 282 mm, FoV phase 76.6%, 192 slices) and fluid attenuated inversion recovery (FLAIR) (TE 353 ms, TR 6000 ms, FoV read 282 mm, FoV phase 85.9%, 192 slices) sequences. Furthermore, diffusion-weighted imaging, resting state fMRI and arterial spin labeling sequences were acquired and will be reported elsewhere.

### Data Processing

Analysis of MRI data was carried out in a blinded manner, which was unblinded for the statistical analysis.

#### Cortical Thickness and Hippocampal Volume

The T1-weighed data was gradient unwarped to correct for spatial distortions (Jovicich et al., 2006) and processed with version 5.3 of the cross-sectional (Fischl et al., 2002) and longitudinal (Reuter et al., 2012) Freesurfer stream, in order to obtain segmentations of cortical regions defined according to the Destrieux atlas (Destrieux et al., 2010) as well as the hippocampal subfields (Van Leemput et al., 2009), caudate and putamen. The pipeline was specifically tuned to correct for intensity inhomogeneity that can be observed at 3T (Boyes et al., 2008; Zheng et al., 2009).

In cases where Freesurfer failed to properly delineate the white matter and pial surface, the pipeline were manually guided following the steps outline in the Freesurfer documentation.^1^ This specifically involved correcting the skull stripping to better delineate the pial surface, insertion of control points to guide white matter normalization for the purpose of improving white matter segmentation, and finally editing the white matter segmentation itself. Two trained readers edited the pipeline; to avoid segmentation bias, one was responsible for skull stripping and white matter editing, while the other was responsible for control point insertion.

Finally, overall quality of the longitudinal segmentation output was assessed by experienced raters blinded to group allocation (CTL, KSF, and EG). Specifically, the pial and white matter surface outlines, as well as the hippocampal subcortical segmentation were visually inspected and consensus reached for all. One volume was excluded due to significant segmentation error in the hippocampus.

To explore regional, cortical effects, cortical thickness measures obtained from Freesurfer were divided into four categories (early, middle, late, and very late) each including areas reported to be progressively affected by atrophy from MCI to full AD diagnosis (Whitwell et al., 2007): “early” (temporal, precuneus, and cingulate), “middle” (parietal, temporal-occipital, occipital, fusiform, and parahippocampus), and “late” (frontal). A “very late” region composed by the pre and postcentral cortex were also defined (**Table 1**).

**Table 1:** Anatomical labels for statistical analysis.

	Regions
Early	Temporal gyri (inferior, medial, superior lateral, transverse, plan-polar, plan-temporal) Precuneus Cingulate
Middle	Parietal gyri (inferior-angular + supramar, superior) Parahippocampal gyrus Fusiform gyrus
Late	Frontal gyrus (inferior-opercular/orbital/triangular, medial, superior)
Very late	Precentral gyrus Postcentral gyrus


*Anatomically labeled cortical regions [according to Destrieux atlas nomenclature (FreeSurfer)] used to construct Early, middle, late and very late regions for statistical analysis.*

#### White Matter Hyperintensities

For delineation of white matter hyperintensities (WMH), MPRAGE and T2-weighted images were co-registered and re-sliced to the corresponding FLAIR image using a six parameter rigid transformation. WMH were defined as clearly hyperintense areas relative to surrounding white matter on both FLAIR and T2-weighted images and identified by simultaneous inspection of both aligned images. For WMH volume local thresholding was applied and WMH volumes for the whole brain quantified automatically using the Jim image analysis package, Version 6.0, (Xinapse Systems Ltd., Northants, United Kingdom)^2^. Visual identification and delineation was carried out by a single trained rater blinded to clinical information. For nine subjects (five control, four intervention) WMH could not delineated due to movement artifacts. WMH volume was used solely as covariate in the statistical analysis.

#### Longitudinal and Normalized Measures

Longitudinal measures of brain volume, cortical thickness and cognitive scores for each subject were computed as the relative change between baseline and follow-up by subtracting baseline from follow-up, and dividing the difference with the baseline measure, thereby canceling out within-subject correlations, as well as accounting for between-subject differences in brain size. Decreases in volume are indicated by negative (-) sign. Throughout the paper, we will refer to the relative change simply as *change*. A normalized WMH measure was obtained by dividing WMH volume with white matter volume.

### Cognitive Outcome Measures

Cognitive assessment included the MMSE for global cognitive impairment (Folstein and Folstein, 1975), the Symbol Digit Modalities Test (SDMT) after 120s for mental speed and attention (Smith, 1973), and the Stroop Color and Word Test (Stroop) (Golden, 1978) incongruent score for reaction time.

### Assessment of Activities of Daily Living

Activities of daily living (ADL) functioning was assessed using the AD Cooperative Study-ADL scale, and is reported for baseline.

### Assessment of Physical Activity Level

The Physical activity scale in the elderly (PASE) (Washburn and Smith, 1993) was used to assess baseline physical activity level. The PASE is a 5-min questionnaire, which asks the participant to recollect activities in the 7 days up until questioning in the domains of work-related, household-related and leisuretime-related physical activity. For the present study, we used proxy-rated PASE scores (only reported for baseline).

### Test of Physical Performance

The 6-min Astrand Cycle Ergometer test (Monark Ergomedic 839E; Monark Exercise AB, Sweden) was used to estimate the maximal oxygen uptake based on workload and average heart rate (HR) during the last minute of the 6-min cycle test, corrected for age and body weight. Maximal oxygen uptake (estimated VO2 max) was used as a measure of aerobic, physical fitness (Cink and Thomas, 1981).

### Exercise Load (Attendance and Intensity)

To assess attendance and intensity of training the physiotherapist in each group kept a training log. Attendance ratio was defined as number of attended exercise sessions over total number of offered sessions. Exercise intensity was based on the per-session average HR recorded using continued monitoring during exercise (including rest). Average HR for all sessions was calculated, and intensity defined as average HR over maximum expected HR (220 minus subject age). To obtain total exercise load, measures for attendance ratio and intensity was multiplied.

### Statistical Analysis

Student’s unpaired *t*-test and the chi squared-test were used to compare baseline demographic and clinical characteristics. To test whether participants who were randomized but were not included in the final analysis differed from those who were included in the analysis, we carried out Student’s unpaired *t*-test to test for differences in age and baseline MMSE and chi squared-test for gender.

#### Brain Volume Measures

Separate multivariable linear regression models were used to compare changes in volume between groups for the hippocampal subfields (model 1), para-hippocampus (model 2), caudate and putamen (model 3) with Bonferroni correction for multiple comparisons. Similarly, separate models were used to compare changes in cortical thickness for each of the “early,” “middle,” “late,” and “very late” categories previously described. For all group tests, Hoteling’s T^2^ multivariate test (Hotelling, 1931) was applied. Since outliers were detected in scatter plots of the variables, a further non-parametric Oja rank sum test (Oja and Randles, 2004) were performed, using 10.000 permutations, to confirm validity of *p*-values from Hoteling’s T^2^ test. Gender, age, and baseline WMH were used as covariates.

#### Correlation Tests

The relationship between changes in frontal and cingulate cortical thickness, and mental speed and attention (SDMT, Stroop) were assessed with pre- and postcentral cortex as a control region, since these regions show no or only very late atrophy in AD patients. The SDMT and Stroop scales were chosen based on findings from previous studies indicating a specific effect of exercise on measures of mental speed and attention (Baker et al., 2010b; Frederiksen et al., 2014), which was also confirmed in the ADEX study with findings of a significant effect of the intervention on SDMT in a subgroup of participants (Hoffmann et al., 2015). Finally, relationship between exercise load, change in estimated VO2 max and changes in hippocampal subfield volume as well as frontal cortical thickness were also investigated. The hippocampus was chosen because hippocampal atrophy is a hallmark pathological finding in AD, and since episodic memory deficits is the main clinical feature of AD. The frontal lobe was chosen due to the aforementioned effect of exercise on mental speed and attention, cognitive functions, which to a high degree are reliant on frontal lobe circuitry. Lastly, to assess whether participants who at baseline had more advanced brain atrophy were less able to participate in the intervention, we examined correlations between exercise load and hippocampal volume and frontal cortical thickness.

An omnibus test was performed on the correlations. The null-hypothesis was that all correlations between the measure of interest and the cerebral volume/thickness measures were zero. In this way the multiple comparison problem was minimized. If the null-hypothesis were rejected, a *post hoc* analysis of the individual correlations was performed.

For all tests, the significance level was 0.05. Statistics were obtained with SAS Statistical Software version 9.4.

## Results

A total of 41 subjects (intervention: 21; control: 20) were included. There were no significant differences between the two groups with regards to age, gender, baseline MMSE, baseline ADL score, or baseline PASE (**Table 2**). There was no significant difference at baseline for participants who dropped out and those who did not regarding baseline MMSE, age and gender. Participants in the intervention group showed an improvement in estimated VO2 max (mean 2.5 mL/kg/min; (SD: ± 3.4); *p* = 0.005) following the intervention, whereas the control group did not (mean 0.2 mL/kg/min; (SD: ± 7.4); *p* = 0.93). Baseline MRI measures are presented in **Table 2**.

**Table 2:** Baseline demographic and clinical data.

	Intervention group	Control group	*P*-value
Age [mean (SD)]	67.8 (±7.7)	69.8 (±7.7)	0.46
Gender (females/male)	9/21	8/20	0.41
MMSE [mean (SD)]	25.7 (±3.6)	27.8 (±2.4)	0.97
SDMT [mean (SD)]	34.8 (12.3)	27.9 (±13.8)	0.11
Stroop [mean (SD)]	18.1 (±11.2)	19.4 (±9.7)	0.72
ADCS-ADL [mean (SD)]	67 (±8.6)	68.3 (±7.1)	0.60
PASE [mean (SD)]	101.4 (±42.7)	96.6 (±46.5)	0.74


*P-values indicate results from Students t-test and Chi-squared where relevant. MMSE, Mini mental state examination; SDMT, Symbol digit modalities test; ADCS-ADL, Alzheimer’s disease cooperative study-Activities of daily living; PASE, Physical activity scale in the elderly.*

### Brain Volumes

In Model 1 (hippocampal subregions), significant difference were found for change between the two groups in the left fimbria (*p* = 0.012) and CA2 + 3 (*p* = 0.016) which did not survive correction for multiple comparison. No difference between groups was observed for Model 2 (parahippocampal) or Model 3 (caudate and putamen). No significant between-group difference in changes in regional cortical thickness was found. See **Table 3**.

**Table 3:** Baseline and change from baseline total hippocampal and subregional volumes.

	Right				Left			
		
	Inter-vention baseline	Change from baseline	Control baseline	Change from baseline	Inter-vention baseline	Change from baseline	Control baseline	Change from baseline
Hippo-campus	2338.5 (±610.0)	-32.2 (193.9)	2226.1 (±390.6)	-50.6 (±218.4)	2199.0 (±452.4)	-42.9 (±127.0)	2007.8 (±424.7)	-15.62 (±145.5)
Presubiculum	292.4 (±56.4)	8.9 (±187.3)	271.8 (±66.2)	-19.4 (±157.2)	305.8 (±60.4)	11.1 (±262.8)	282.4 (±74.6)	8.1 (±195.7)
Subiculum	242.1 (±42.0)	-18.6 (±139.7)	247.3 (±51.3)	-2.5 (±150.9)	244.3 (±43.8)	-28.1 (±162.9)	238.1 (±56.7)	21.4 (±133.1)
Fimbria	679.1 (±141.4)	-1.4 (±51.6)	640.7 (±113.0)	-6.8 (±78.6)	656.2 (±122.2)	-17.6 (±59.5)	594.1 (±133.0)	-9.6 (±84.7)
CA1	29.5 (±17.9)	22.5 (±191.4)	67.1 (±19.2)	11.8 (±174.4)	40.3 (±22.0)	-1.1 (±114.3)	33.4 (±19.1)	-24.5 (±170.2)
CA2_3	412.6 (±81.0)	11.3 (±236.8)	381.7 (±82.2)	-24.0 (±331.1)	419.9 (±83.1)	-10.6 (±266.7)	387.0 (±88.7)	22.8 (±278.1)
CA4_ DG	381.3 (±82.6)	-41.4 (±135.5)	357.2 (±66.1)	-32.0 (±135.8)	372.6 (±72.4)	-28.7 (±162.9)	344.1 (±73.4)	17.0 (±151.1)


*Volumes are in mm^*3*^ and reported as mean (SD). Anatomical labels refer to those applied in the FreeSurfer atlas. CA, Cornu Amonis; DG, Dentate gyrus.*

### Cognitive Performance Correlations

**FIGURE 2:**
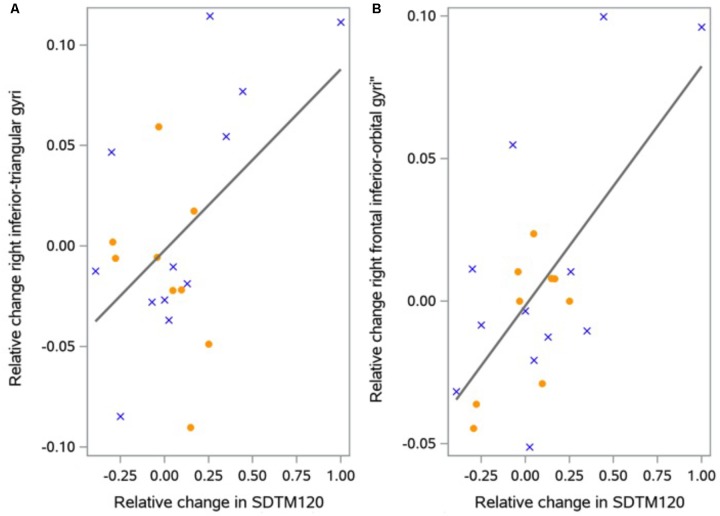
Correlation between change in cortical thickness and change in SDMT. The figure shows correlations between relative change in inferior-triangular gyri **(A)** and right frontal inferior-orbital **(B)** thickness and relative change in SDMT (Unitless) for each region in the exercise group. Female participants are indicated by solid circles, males by crosses.

Relative change in frontal and cingulate cortical thickness correlated significantly with change in SDMT (*p* = 0.0078) in the intervention group. Specifically, for frontal area in the intervention group, a *post hoc* analysis revealed change in cortical thickness in the right frontal inferior-orbital gyri (*r* = 0.48, *p* = 0.038) and right frontal inferior-triangular gyri (*r* = 0.64, *p* = 0.003) (**Figure 2**) to be significantly correlated with change in SDMT. Change in cortical thickness and SDMT did not correlate in the usual care group. When combining the two groups, there was a significant correlation [right frontal inferior-orbital gyri: *r* = 0.46, *p* = 0.004; right frontal inferior-triangular gyri (*r* = 0.38, *p* = 0.02)]. There were no significant correlations between change in cortical thickness and Stroop scores (*p* = 0.08).

### Exercise Load and Physical Performance Correlations

In the intervention group, exercise load was correlated with change in frontal cortical thickness (*p* = 0.011), with a *post hoc* analysis revealing right inferior frontal area to be significantly correlated with exercise load (*r* = 0.51, *p* = 0.035). Similarly, a significant correlation with changes in hippocampal volume (*p* = 0.009) was found, with *post hoc* analysis revealing no individually significant areas, but with the largest correlation in the right subiculum (*r* = 0.44, *p* = 0.086). There was no significant correlation between hippocampal subfield volume and exercise load or between hippocampal volume and frontal cortical thickness and estimated VO2 max. There was no correlation between baseline hippocampal volume or frontal cortical thickness and exercise load.

## Discussion

The effect of physical exercise on hippocampal volume has only been reported in a small number of studies, but nevertheless it remains the most consistent finding. Other brain regions have been reported to be influenced by exercise, including whole brain volume (Mortimer et al., 2013), anterior and posterior cingulate gyrus (Scheewe et al., 2013; Reiter et al., 2015; Köbe et al., 2016), parietal cortical area (Taubert et al., 2011; Köbe et al., 2016), insula (Reiter et al., 2015), and precentral gyri (Reiter et al., 2015). Another region, which has been reported to be positively affected by exercise interventions in several studies, is the frontal cortical region (Taubert et al., 2011; Scheewe et al., 2013; Reiter et al., 2015; Tamura et al., 2015; Köbe et al., 2016). Although we were not able to show a similar effect, we found change in SDMT and exercise load to be correlated with relative change in frontal cortical thickness. In the main ADEX study population totaling 200 patients with AD, we have previously reported a positive effect on SDMT in a subpopulation of participants who adhered to the exercise program (Hoffmann et al., 2015). Our findings in the present study could be interpreted to indicate that changes in frontal cortical thickness may be the underlying biology mediating the effect on SDMT. However, such speculation must be made with caution since the present correlation was in a relatively small group and since correlations can only imply, but not prove a causative relationship. It is also not surprising that changes in frontal regions may be associated with change in SDMT, primarily a measure of mental speed and attention, which rely on frontal circuitry. Furthermore, the fact that several studies report effects of exercise in frontal regions may also simply be an effect of the relative size of the frontal lobe. Nevertheless, other studies have found cognitive functions subserved by frontal areas (e.g., attention, executive function) to be more amenable to effects of exercise, relative to e.g., memory function (Baker et al., 2010b; Frederiksen et al., 2014). The correlation between exercise load and change in frontal cortical thickness may be interpreted as an indication of a dose-response relationship or possibly a responder effect where the effect of the intervention is confined to those individuals who were the most active. From a biological standpoint, it would be a reasonable assumption, and is seemingly supported by the previously mentioned findings regarding SDMT in the subpopulation in the ADEX study (Hoffmann et al., 2015). However, the lack of a correlation between change in frontal cortical thickness and physical fitness (measured by estimated VO2 max), which may be a more accurate measure of the effects of the intervention, contradicts such an interpretation. Rather, it could be speculated that the significant correlation indicates a reverse relationship, i.e., that participants with less pronounced brain atrophy, were able to participate more in the exercise intervention. We however, also tested this hypothesis by examining correlations between hippocampal volume and frontal cortex and exercise load, and found no evidence for this reverse relationship.

The study has several limitations, which must be taken into account when interpreting the presented findings. Our study population was relatively small, with a total of 41 participants. Moreover, the duration of the intervention was short compared to some other studies, in which interventions up to 2 years have been applied (Tamura et al., 2015), the most common being 6–12 months (Colcombe et al., 2006; Erickson et al., 2011; Niemann et al., 2014; Best et al., 2015). There is a tendency for studies, which reported significant effects on brain volumes to apply interventions between 6 and12 months of moderate intensity (target of 60–70% of maximal HR) (Erickson et al., 2011; Mortimer et al., 2013; Scheewe et al., 2013; Niemann et al., 2014; Best et al., 2015; ten Brinke et al., 2015), indicating a threshold of around 6 months as a minimum to elicit detectable changes. In this context it is noteworthy that the exercise intervention in the present subgroup as well as the ADEX cohort as a whole, was able to improve aerobic fitness in participants (Sobol et al., 2016). Further, in the main ADEX study, an improvement in SDMT in a subgroup, and fewer neuropsychiatric symptoms in the intervention group, was found, demonstrating target engagement regarding these measures. The present finding do not clearly support detectable structural brain changes to underlie these improvements in clinical measures, although they cannot be interpreted as negating such a relationship.

One study examined an intervention of 19 weeks in patients with stroke. 18F-FDG-PET scans were used to assess regional brain glucose metabolism, a marker of synaptic function. The authors reported a significant increase in glucose metabolism in the medial temporal lobe, but not a concomitant effect on MRI derived measure of cortical atrophy (Moore et al., 2015). These findings highlight the issue of whether the applied method of MRI may be less sensitive compared to other methods to detect changes induced by exercise in brain pathophysiology and normal physiology, especially with regards to shorter interventions. Other potentially more sensitive imaging modalities include resting state fMRI, MR spectroscopy, perfusion MRI and ultra-high field MRI, but studies are lacking and as such remain speculative. These imaging modalities should be explored in future studies. Moreover, results from animal models of aging and AD have shown that many biological mechanisms may be responsible for the brain-exercise relationship, such as neurogenesis (van Praag et al., 1999; Intlekofer and Cotman, 2013), angiogenesis (Paillard et al., 2015) and reduction of beta-amyloid (Yuede et al., 2009) which are not readily quantified by MRI.

The methodology of our study also has several strengths. As described elsewhere (Reuter et al., 2012), the longitudinal Freesurfer pipeline utilizes cross-sectionally processed time points to generate a common template, which is then used as a point of initialization for an unbiased analysis of each individual timepoint. This procedure helps to avoid potential bias in the outcome measures due to e.g., registration to the baseline time point, as pointed out in (Fox et al., 2011). Furthermore, it increases statistical power because inter-subject variation is reduced. The ADEX study was conducted following a rigorous methodology regarding planning of the study, randomization procedures and assessment of attendance and intensity of exercise. Study participants were well characterized with regards to diagnosis, clinical and demographic data, cognitive performance and aerobic fitness. In this subgroup, this included biomarkers, which confirmed the presence of AD pathology such as beta-amyloid of phosphorylated-tau (i.e., amyloid PET and CSF sampling). Furthermore, we conducted a pilot study prior to the main study to be able to design an exercise program specifically designed to patients with dementia (64). In the present study, a drop-out rate of 18 % was found (compared to 10 percent in the whole ADEX cohort (Hoffmann et al., 2015). For the main ADEX study, a drop-out rate of 20 % was expected (Hoffmann et al., 2013), and as such, the drop-out rate is less than expected. In a systematic review of intervention studies in AD, drop-out rates of 22–51% (for placebo 22–41%) was found (Grill et al., 2017), and for exercise interventions, drop-out rates between 10–20% have previously been reported (Rolland et al., 2007; Santana-Sosa et al., 2008; Eggermont et al., 2009; Baker et al., 2010b; Yu et al., 2011).

## Conclusion

In conclusion, a 16-week intervention of moderate-to-high intensity aerobic exercise was unable to attenuate atrophy rates in patients with mild to moderate AD. The intervention period may not have been long enough for target engagement with regards to MRI measures of gray matter atrophy, although patients in the intervention group improved with regards to aerobic fitness. In this context, it is pertinent to highlight the fact that even though the present study failed to find significant effects on cortical volume, we have shown that the 16-week exercise intervention did improve symptoms in the participants (Hoffmann et al., 2015). This has practical implications in that it underscores the need to offer exercise regimes even of shorter duration (e.g., due to restricted resources or inability of patients to participate for longer periods) to patients with dementia. Correlations between frontal cortical thickness and exercise load and a measure of mental speed and attention, are in line with previous findings of an effect on the frontal lobe, and functions subserved by this brain region. Further studies in AD populations applying rigorous methodology and longer interventions of more than 6 months are needed to elucidate effects on brain volume.

## Data Availability

The raw data supporting the conclusions of this manuscript will be made available by the authors, without undue reservation, to any qualified researcher.

## Author Contributions

KF and CL contributed to the design and data collection of the ADEX study and the present study, gave input to the statistical analysis of data, and drafted the manuscript. SH contributed to the design and data collection of the ADEX study and the present study, and was a major contributor to the paper. AC planned and carried out the statistical analysis of data and was a major contributor to the paper. PH contributed to the design and data collection of the ADEX study and the present study, and was a major contributor to the paper. LW contributed to the design and data collection of the ADEX study and the present study, and was a major contributor to the paper. BA contributed to the design and data collection of the ADEX study and the present study, and was a major contributor to the paper. HS contributed to the design of the present study, and was a major contributor to the paper. EG contributed to the design of the present study, and was a major contributor to the paper. All authors read and approved the final manuscript. The corresponding author takes primary responsibility for communication with the journal and editorial office during the submission process, throughout peer review, and during publication. The corresponding author takes responsibility for ensuring that the submission adheres to all journal requirements including, but not exclusive to, details of authorship, study ethics and ethics approval, clinical trial registration documents, and conflict of interest declaration. The corresponding author will be available post-publication to respond to any queries or critiques.

## Conflict of Interest Statement

The authors declare that the research was conducted in the absence of any commercial or financial relationships that could be construed as a potential conflict of interest.

## Footnotes

1 http://freesurfer.net/fswiki/FreeSurferWiki

2 www.xinapse.com
